# Ileo-Colon Targeting of the Poorly Water-Soluble Drug Celecoxib Using a pH-Dependent Coating in Combination with Self-Emulsifying Drug Delivery or Solid Dispersion Systems

**DOI:** 10.3390/pharmaceutics13050731

**Published:** 2021-05-15

**Authors:** Annemarie Broesder, Julia M. E. Berends, Sophie M. Scheepers, Duong N. Nguyen, Henderik W. Frijlink, Wouter L. J. Hinrichs

**Affiliations:** Groningen Research Institute of Pharmacy, Department of Pharmaceutical Technology and Biopharmacy, University of Groningen, Antonius Deusinglaan 1, 9713 AV Groningen, The Netherlands; a.broesder@rug.nl (A.B.); j.m.e.berends@student.rug.nl (J.M.E.B.); sophiescheepers@gmail.com (S.M.S.); nnduong19@gmail.com (D.N.N.); h.w.frijlink@rug.nl (H.W.F.)

**Keywords:** ileo-colonic drug delivery, supersaturation, film coating, BCS class II drug, delayed release, ColoPulse

## Abstract

Targeting celecoxib to the ileo-colonic region could be beneficial for the treatment and prevention of colon cancer. Ileo-colonic targeting can be achieved by using pH-dependent coating systems such as ColoPulse. Celecoxib has poor aqueous solubility, which may jeopardize optimal treatment. Therefore, we combined a pH-dependent coating with self-emulsifying drug delivery systems (SEDDS) or with solid dispersion systems (SD); two approaches that are often used to improve the dissolution behavior of lipophilic drugs. The dissolution behavior of various formulations of both systems was investigated. Optimized formulations with and without precipitation inhibitors were coated with the ColoPulse and the release of celecoxib was tested under non-sink conditions using an in vitro dissolution system, simulating the pH gradient of the gastrointestinal tract. The dissolution behavior of SDs with and without precipitation inhibitor (sodium dodecyl sulfate) and the SEDDS without precipitation inhibitor was negatively impacted by the coating. Control experiments indicated that components of the coating released in the dissolution medium acted as precipitation mediators. However, the SEDDS formulation with HPMC 4000 cps as a precipitation inhibitor showed excellent dissolution behavior. We hypothesize that HPMC accumulates at the oil/water interface of the emulsion thereby stabilizing the emulsion resulting in maintenance of the supersaturated state.

## 1. Introduction

Celecoxib (CXB) has shown promising results in the treatment and prevention of colon cancer [1,2]. CXB is a selective inhibitor of Cyclooxygenase-2 (COX-2). COX-2 has been found to be overexpressed in cancer tissue and inhibition of COX-2 has been found to promote apoptosis and reduce cell proliferation in cancer tissues, e.g., colorectal cancer [1,2]. Lemmens et al. found that orally dosed CXB was partially absorbed in the small intestine and the unabsorbed CXB accumulated in the colonic tissue [3]. This study indicates that CXB probably does not exert its therapeutic effect on colon cancer via the systemic circulation. The systemic absorption of CXB can lead to cardiovascular side effects [4]. To reduce these systemic side effects and to obtain higher drug concentrations at the site of action with lower dosages, targeted drug delivery to the colon may be beneficial.

CXB belongs to the biopharmaceutics classification system (BCS) class II drugs, i.e., it has low solubility (3–7 mg/L at pH 7) and high permeability (log P 3.5) [2,5]. This low solubility could lead to a dissolution limited drug release in the colon, which may reduce the efficacy of CXB. Two frequently used strategies to improve the dissolution behavior of BCS class II drugs are solid dispersions (SD) and self-emulsifying drug delivery systems (SEDDS) [6]. Thus, it is not surprising that in various studies CXB has been incorporated in SD and SEDDS formulations [7,8,9,10].

To obtain ileo-colonic drug delivery, researchers have utilized the pH gradient in the gastrointestinal tract, bacterial degradation in the colon, transit time, and changes in intestinal pressure [11]. Two formulation strategies, which utilize one of the aforementioned triggers for passive targeting, are matrix systems and film coated systems. The ColoPulse coating system is designed to obtain ileo-colonic drug delivery [12]. The coating, which is based on the pH-sensitive polymer Eudragit S100, disintegrates in the terminal ileum due to a local pH peak above 7.2 [13]. There are numerous studies which use Eudragit S100 to passively target to the ileo-colonic region, e.g., coated liposomes [14], nanoparticles [15], and dual coated systems [16,17]. The ColoPulse coating differentiates itself by the incorporation of a superdisintegrant in the Eudragit matrix in a non-percolating manner. The local pH peak above 7.2 results in the dissolution of Eudragit S100 exposing the superdisintegrant particles in the coating to an aqueous environment in which they strongly swell resulting in the rapid disintegration of the coating. The ColoPulse coating could thus be used to target CXB to the ileo-colonic region. Therefore, the objective of this study was to investigate which strategy, SD or SEDDS, can best be used for CXB in combination with the ColoPulse coating. This is of importance since the influence of a coating system on the release profile could alter the decisions made in the early development of formulations for BCS class II drugs. In this study, we show that SD and standard SEDDS formulations of CXB are negatively impacted by the ColoPulse coating while a supersaturated SEDDS formulation was not impacted and maintained the supersaturated state. 

## 2. Materials and Methods

### 2.1. Materials

Celecoxib (CXB), Eudragit S100, and hydroxypropyl methylcellulose acetate succinate LG (HPMCAS-LG) were supplied by Jansen Pharmaceutica (Beerse, Belgium). Capryol 90, Maisine CC, Peceol, Plurol oleique CC 497, and Labrasol were a gift from Gattefossé (Saint-Priest Cedex, France). Captex 200 P, Captex 355 EP/NF, and Capmul PG-8 NF were a gift from ABITEC Corporation (Columbus, OH, USA). Croscarmellose sodium (AcDiSol) was obtained from FMC BioPolymer (Philadelphia, PA, USA). Tween 20, isopropyl myristate, Cremophor RH40, PEG 400, tetraglycol, Patent Blue V, Povidone (PVP) K15, PVP K60, and PVP K90 were obtained from Sigma-Aldrich (St. Louis, MO, USA). Gelatine Licaps^®^ size 0 capsules were gifted by Capsugel (Bornem, Belgium). Macrogolum 6000 (PEG 6000), PVP K30, hydroxypropyl methylcellulose (HPMC) 5 and 4000 cps, talc, and gelatine were obtained from BUFA (IJsselstein, The Netherlands). Tween 80, 37% fuming hydrochloric acid, sodium chloride (NaCl), sodium dodecyl sulfate (SDS), tert-butyl alcohol (TBA), sodium dihydrogen phosphate dihydrate, and sodium hydroxide (NaOH) were obtained from MERCK (Darmstadt, Germany). Acetone and ethanol 70% were purchased from BOOM B.V. (Meppel, The Netherlands) and methanol from VWR Chemicals (Fontenay-sous-Bois, France). Millipore type 1 water was used in all experiments except for the dissolution media where demineralized water was used. 

### 2.2. Gastrointestinal Simulation System

To test the performance of the ColoPulse coating in vitro, the gastrointestinal simulation system (GISS) was used. The GISS is a dissolution test that simulates the pH profile of the gastrointestinal tract in four distinct phases [18]. The different phases of the GISS are given in Table 1. Originally the phosphate buffer used in the GISS was a potassium salt. However, due to the incompatibility of SDS, which was incorporated into some of the SD formulations, with potassium ions, the potassium salt in the GISS was substituted with a sodium salt. 

### 2.3. SEDDS

#### 2.3.1. Saturation Concentration

To assess the saturation concentration of CXB in different oily phases (Capryol 90, Captex 200 P, Captex 355 EP/NF, isopropyl myristate, Maisine CC, and Peceol), surfactants (Cremophor RH40, Labrasol, Tween 20, and Tween 80), and co-surfactants/co-solvents (Capmul PG-8 NF, PEG 400, Plurol oleique CC 497, and tetraglycol), an excess amount of CXB was added to the liquid excipients and placed in a tube rotator at 20 rpm and 30 °C for a day. After that, samples were centrifuged at 14,000 rpm for 20 min and the supernatant was appropriately diluted in methanol. The drug concentration was determined at 252 nm with a UV-VIS spectrophotometer Unicam UV 500A (Gemini, Apeldoorn, The Netherlands). The concentration was measured again every two to three days until it did not change anymore, thus assuming that the saturation concentration was reached.

#### 2.3.2. Solubilization Capacity

The surfactants and co-surfactants/co-solvents with the highest saturation concentration of CXB were further screened to determine their solubilization capacity for the oily phase [19]. The oily phase with the highest CXB solubility was used in these experiments. To 10 mL of a 10% *w*/*v* surfactant solution in water, 10 µL oily phase was added and the mixture was vortexed for 30 s. The transparency of the solution was judged by visual inspection after 4.5 min. If clouding did not occur another 10 µL oily phase was added and re-analyzed. This procedure was repeated until clouding was observed. The same technique was used to evaluate the optimal co-surfactant/co-solvent combination. However, 10 mL of a 5% *w*/*v* co-surfactant/co-solvent with 5% *w*/*v* of the selected surfactant in water was used.

#### 2.3.3. Pseudo Ternary Phase Diagram

A pseudo ternary phase diagram in the presence of CXB was made of the selected oily phase, surfactant, and co-solvent/co-surfactant. For the phase diagram, the surfactant to co-solvent/co-surfactant volume ratio (S_mix_) was kept constant at 1:1. The following S_mix_ to oil volume ratios were used: 1:9, 2:8, 3:7, 4:6, 5:5, 6:4, 7:3, 8:2, and 9:1. The S_mix_:oil mixtures contained 20 mg/mL CXB. Water was added to the S_mix_:oil mixtures in 500 µL Eppendorf tubes to a final volume percentage varying from 5% to 95% and an end volume of 500 µL. Directly after the addition of water, the samples were vortexed for 30 s and stored at room temperature for 24 h. After 24 h, the solutions were visually examined against a black background for transparency and phase separation. Separated phases indicated the formation of an unstable emulsion, turbidity or a white appearance indicated the formation of a stable emulsion, and transparency or light bluish appearance indicated the formation of a micro-emulsion/nano-emulsion. The classification according to the appearance was based on Cui et al. [20]. The phase diagram was constructed with the aid of Triplot software version 4.1.2 (Todd A. Thompson, Indiana University, Bloomington, IN, USA).

#### 2.3.4. Dynamic Light Scattering

The droplet size of the final formulations was measured with dynamic light scattering (Mobius, Wyatt Technology, Santa Barbara, CA, USA) at different dilutions: 2000×, 4000×, and 8000× in GISS phase III in triplicate. GISS phase III was filtered through a 0.02 µm filter before the SEDDS was immersed into it. An acquisition time of 5 s and an acquisition number of 10 were used. The samples were measured within 10 min upon dilution. The droplet size was obtained by regularization analysis in the DYNAMCIS Software (Wyatt Technology Corporation, Santa Barbara, CA, USA) since it is appropriate for both monomodal and multimodal samples. Results were statistically analyzed by using a two-way ANOVA test, *p* values < 0.05 were considered significant.

#### 2.3.5. SEDDS Filled Capsules for Formulation Optimization

To determine the optimal S_mix_:oil volume ratio and precipitation inhibitor different formulations, see Table 2, were filled in capsules. Licaps size 0 capsules were filled with 500 µL SEDDS formulation with S_mix_:oil volume ratios of 7:3, 8:2, and 9:1 with 20% *w*/*v* CXB (thus 100 mg CXB per capsule). To the formulation with a S_mix_:oil volume ratio of 9:1 with 10% *w*/*v* CXB (thus 50 mg CXB per capsule), different precipitation inhibitors were added at a concentration of 2% *w*/*v*: HPMC 5 cps, HPMC 4000 cps, HPMCAS-LG, PVP K15, PVP K30, PVP K60, or PVP K90. All precipitation inhibitors dissolved in the SEDDS formulation except for HPMC 4000 cps which remained a suspension. The CXB dose was lowered from 100 mg to 50 mg in the experiments with the precipitation inhibitor to ensure full dissolution of CXB. The capsules that were used for the formulation optimization were sealed with 70% ethanol with Patent Blue V as a coloring agent. 

### 2.4. Solid Dispersion

#### 2.4.1. Freeze-Drying

The following carriers were screened for the SD formulation: inulin, mannitol, and PVP K30. The carriers were dissolved in Millipore water at a concentration of 75 mg/mL. Other excipients were added to the carrier solution in amounts relative to the amount of carrier, as listed in Table 3. CXB was dissolved in TBA at a concentration of 25 mg/mL. The CXB solution and carrier solutions were mixed in 20 mL Fiolax^®^ clear glass vials (Schott, Mitterteich, Germany) with a maximum volume of 2 mL per vial. The volume ratio of the CXB solution and carrier solution was adjusted to obtain 22.5% *w*/*w* CXB in all freeze-dried powders. Immediately after mixing, the mixtures were snap-frozen in liquid nitrogen and subsequently freeze-dried in a Christ Model Epsilon 2–4 LSCplus freeze-drier (Salm and Kipp, Breukelen, The Netherlands). Freeze drying was conducted using a three-step process. In the first step, the pressure was set at 0.220 mbar and the shelf temperature at −35 °C. Subsequently, the pressure was reduced to 0.050 mbar and the shelf temperature was gradually increased to 25 °C during approximately 16 h. Thereafter, freeze drying was continued for another day at constant pressure of 0.050 mbar and a shelf temperature of 25 °C. During the whole process, the condenser temperature was −85 °C. After collection, the SDs were stored under dry nitrogen. 

#### 2.4.2. X-ray Powder Diffraction

X-ray powder diffraction (XRPD) analyses were performed using a D2 Phaser (Bruker, Billerica, MA, United States of America), equipped with a LynxEye 1D detector and a copper X-ray source, generating radiation with a wavelength of 1.54184 Å. The instrument operated at a voltage of 30 kV and a current of 10 mA using a divergence slit of 1 mm and an air scatter screen of 3 γ/n. The powder was filled into a low background sample holder (C79298A3244B261, Bruker). The diffraction of the samples was recorded at 2θ angles within a scan range of 5 to 40°, at a scan speed of 1 s/step, with a step size of 0.004° and a rotating speed of 60 rpm of the sample stage. 

#### 2.4.3. SD Filled Capsules for Formulation Optimization

To be able to fill a dosage of 50 mg CXB in size 0 gelatin capsules, the density of the freeze-dried powders was increased by dry granulation. The powders were filled into a die with a diameter of 5 cm and compacted at a pressure of 10 kN at a compaction rate of 0.5 kN/s using the 5969 Universal Testing System (Instron, Norwood, MA, USA) equipped with a 50 kN load cell. Subsequently, the compact was granulated using an oscillating granulator AR400 (Erweka, Heusenstamm, Germany) equipped with a 0.8 mm sieve. The capsules that were used for the formulation optimization were not sealed.

### 2.5. Dissolution Test

The capsules filled with either the SEDDS or SD formulation were tested in a USP dissolution apparatus type 2 (Sotax AT 7, Sotax, Basel, Switzerland) with 1000 mL medium at 37 °C and a paddle speed of 100 rpm under non-sink conditions. In-house-made capsule sinkers were used to prevent floating of the capsules. An in-line UV-spectrophotometer (Evolution 300 UV–VIS spectrophotometer, Thermo Fisher Scientific, Madison, WI, USA) measured the absorbance every 3 min at 255 nm for 6 h. The SEDDS 7:3, SEDDS 8:2, and SEDDS 9:1 formulations were tested in demineralized water and the other formulations in GISS phase III as the dissolution medium. 

### 2.6. Influence of the ColoPulse Coating

#### 2.6.1. Capsule Filling for ColoPulse Coating

In experiments regarding the effects of the ColoPulse coating, the SDs were sieved over a 0.5 mm screen sieve after granulation to obtain granules with a narrow size distribution (about 0.5–0.8 mm). Physical mixtures (PM) used in these experiments were filled into the capsules without dry granulation. The capsules filled with the SDs, PMs, and SEDDS with a dose of 50 mg CXB were sealed with a 40% *w*/*w* gelatin solution at 60 °C.

#### 2.6.2. Capsule Coating

The ColoPulse coating consisted of Eudragit S100:PEG6000:AcDiSol:Talc in a weight ratio of 7:1:3:2 in 150 mL acetone:water with a volume ratio of 49:1 [12]. Batch sizes of 30 capsules were spray coated in a mini-rotating drum at 32 rpm with a spray rate of 3.3 mL/min using a peristaltic pump (Minipuls 3, Gilson, Viliers le Bel, France) connected to a nozzle with a bore diameter of 1 mm (Schlick 970, Düsen-Schlick, Coburg, Germany). The temperature was maintained within 20–25 °C with a heat gun. After the coating was applied the capsules were dried in the drum for 5 min. A coating thickness of 19–20 mg/cm^2^ was applied.

#### 2.6.3. Dissolution Test in GISS

The performance of the coated capsules was tested under non-sink conditions in GISS phases I–IV, see Table 1. The same experimental settings were used as described in Section 2.4. Control experiments were carried out to test the release profile of the uncoated capsules in phase III–IV of the GISS. Further, the influence of dissolved ColoPulse coating in GISS phase III–IV was determined by spiking phase III of the dissolution medium with the ColoPulse suspension before the dissolution test. 

## 3. Results

### 3.1. SEDDS

**Figure 1:**
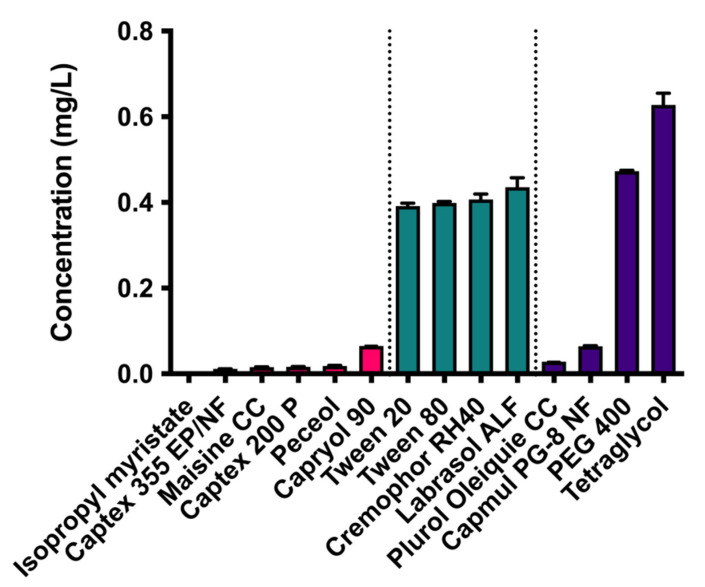
Solubility in mg/mL of CXB in different oils (pink), surfactants (green), and co-surfactant/co-solvent (purple) at 30 °C (mean ± SD, *n* = 3).

**Figure 2:**
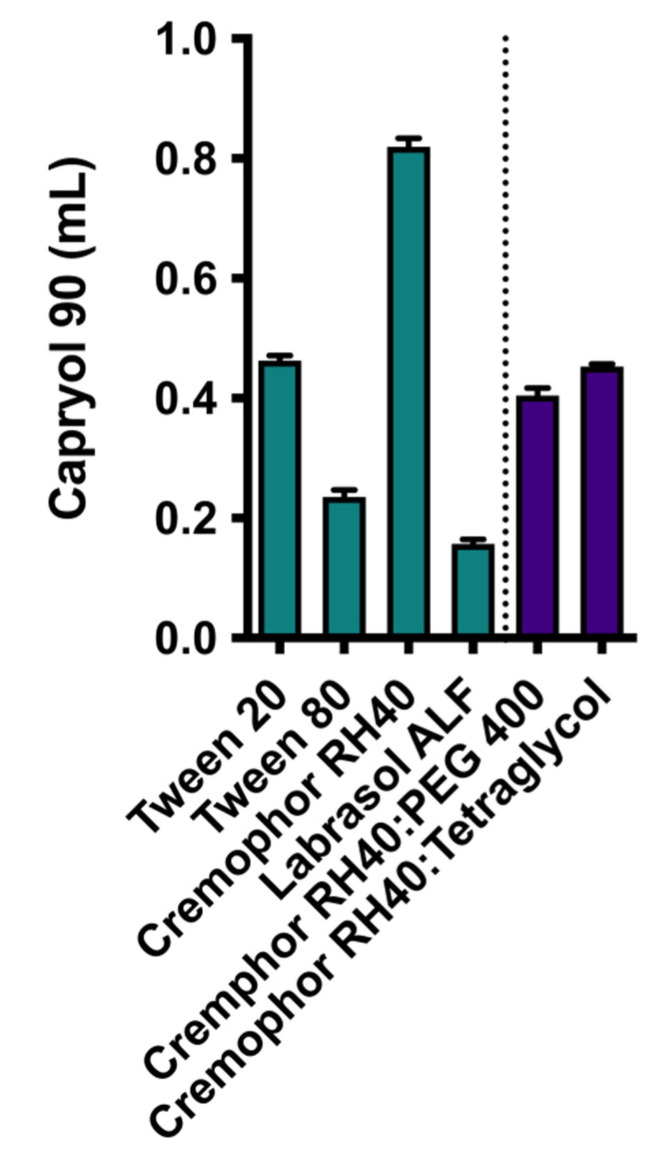
Solubilizing capacity of different surfactants (green) and co-surfactants/co-solvents (purple). The added volume of capryol 90 until the clouding point is given (mean ± SD, *n* = 3).

**Figure 3:**
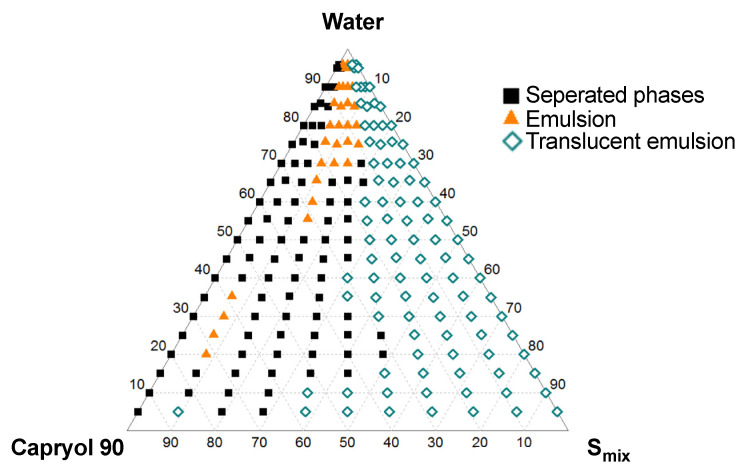
Pseudo ternary phase diagram with capryol 90 as the oily phase and Cremophor RH40:tetraglycol at a volume ratio of 1:1 (S_mix_) as the co-surfactant/co-solvent mixture with 20 mg/mL CXB at ambient temperature.

**Figure 4:**
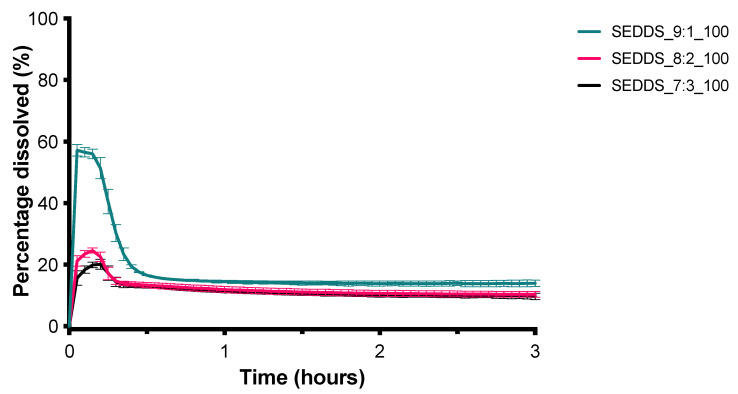
Influence of SEDDS composition on the dissolution profile of SEDDS containing 100 mg CXB in 1000 mL demi water (mean ± SD, *n* = 3).

In Figure 1 the solubility of CXB in the different excipients is given. From the six oily phases evaluated, capryol 90 was able to dissolve the highest amount of CXB and was therefore selected as the oily phase in further experiments. All four surfactants under evaluation, i.e., Tween 20, Tween 80, cremophor RH40, and Labrasol ALF, had comparable CXB solubilities and were further screened for their solubilization capacity of caproyol 90 (Figure 2). Cremophor RH40 showed the highest solubilization capacity and was therefore selected as a surfactant for the SEDDS formulation. From the co-surfactants/co-solvents, PEG 400 and tetraglycol showed the highest solubility of CXB (Figure 1). Tetraglycol was able to solubilize a slightly higher amount of capryol 90 in the presence of cremphor RH40 and was therefore selected as co-solvent (Figure 2). To elucidate the optimal S_mix_ to oil volume ratio, a pseudo ternary phase diagram was made, see Figure 3. The diagram shows that the prevention of phase separation upon dilution is achieved at S_mix_ to oil ratios of at least 7:3. Therefore, the formulations SEDDS_7:3_100, SEDDS_8:2_100, and SEDDS_9:1_100 composed of the S_mix_ to oil volume ratios of 7:3, 8:2, and 9:1 were further screened in dissolution studies, see Figure 4. 

**Figure 5:**
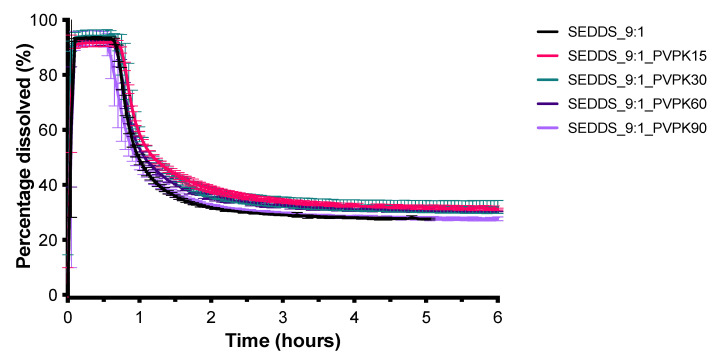
Influence of PVPK15, PVPK30, PVPK60, PVPK90 on the dissolution profile of SEDDS containing 50 mg CXB in 1000 mL GISS phase III (mean ± SD, *n* = 3).

**Figure 6:**
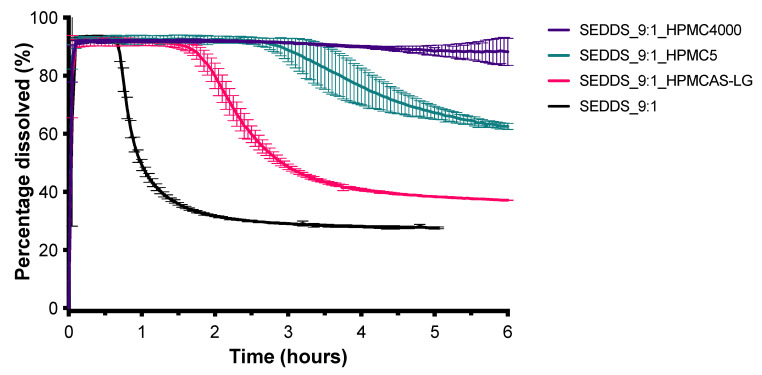
Influence of HPMC 4000 cps, HPMC 5 cps, and HPMCAS-LG on the dissolution profile of SEDDS containing 50 mg CXB in 1000 mL GISS phase III (mean ± SD, *n* = 3).

**Figure 7:**
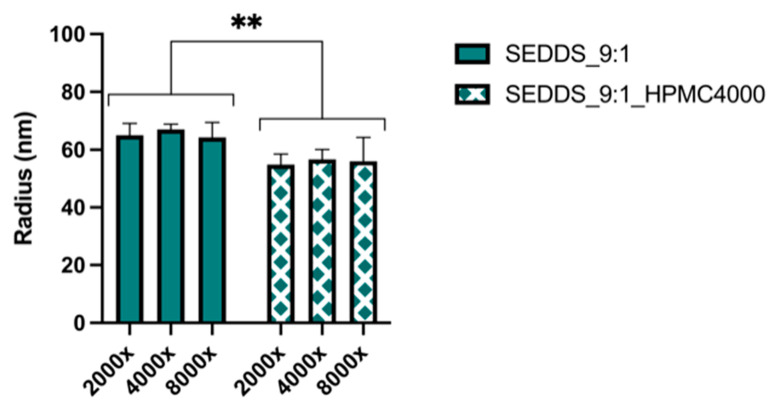
Droplet size SEDDS_9:1 and SEDDS_9:1_HPMC4000 after immersion in GISS phase III at different dilution factors i.e., 2000×, 4000×, and 8000× (mean ± SD, *n* = 3; ** *p* < 0.01).

The SEDDS_9:1_100 formulation which has the highest S_mix_ to oil volume ratio (9:1) resulted in the highest CXB concentration of approximately 55 mg/L, within a few minutes. After about 15 min, however, the CXB concentration rapidly decreased due to precipitation. To test whether the precipitation inhibitors could maintain the supersaturated state for a longer period of time, the dose was lowered to 50 mg (SEDDS_9:1) to obtain a complete release of CXB initially. As shown in Figure 5, the four different types of PVP were not able to slow down the precipitation of CXB. In contrast, all three types of HPMC under investigation were able to do so. HPMC 4000 cps performed the best (Figure 6). The influence of HPMC 4000 cps on the droplet size of the SEDDS_9:1 formulation after water immersion was determined by DLS measurements. As can be seen in Figure 7, the addition of HPMC 4000 cps to the formulation (SEDDS_9:1_HPMC4000) resulted in a significantly smaller droplet size (*p* = 0.0012). Since the concentration of HPMC 4000 cps after dilution ranged from 2.5–10 mg/L, any influence of viscosity on the droplet size was expected to be negligible. This is also evidenced by the fact that the dilution of the samples did not significantly influence the droplet size. 

### 3.2. SD

**Figure 8:**
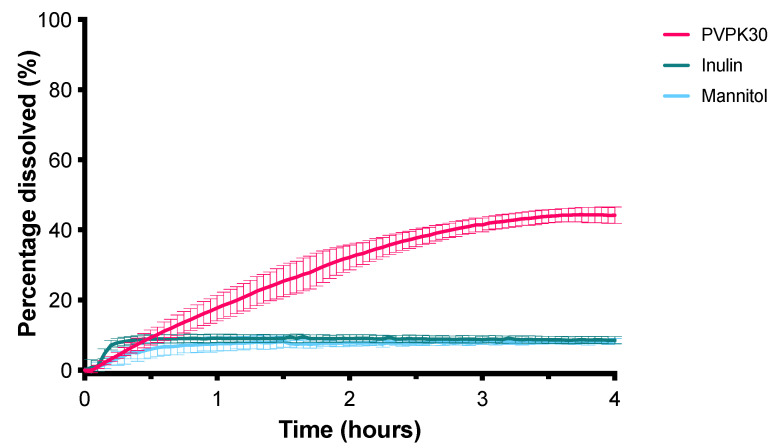
Influence of different carriers on the dissolution profile of SDs containing 50 mg CXB in 1000 mL GISS phase III (mean ± SD, *n* = 3).

**Figure 9:**
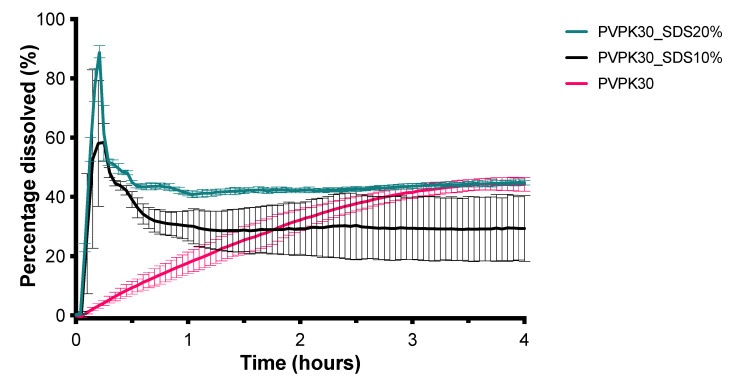
Influence of SDS on the dissolution profile of SDs containing 50 mg CXB in 1000 mL GISS phase III (mean ± SD, *n* = 3).

**Figure 10:**
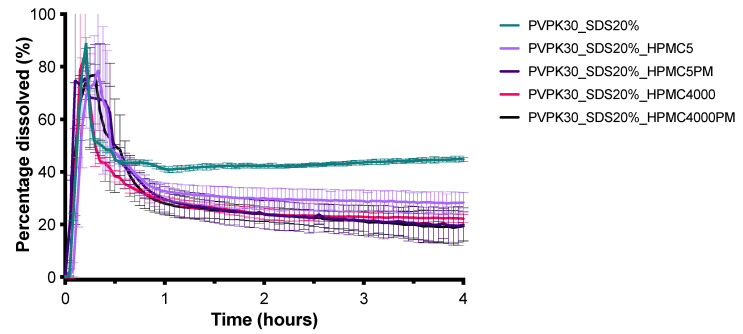
Influence of co-freeze dried or physically mixed HPMC 5 and 4000 cps on the dissolution profile of SDs containing 50 mg CXB in 1000 mL GISS phase III (mean ± SD, *n* = 3).

**Figure 11:**
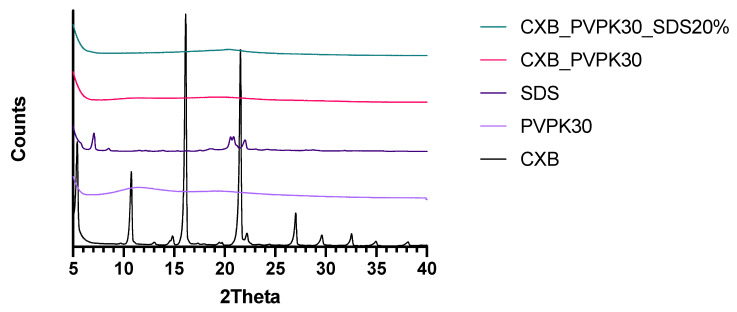
XRPD spectra of CXB, PVPK30, SDS, CXB_PVP30, and CXB_PVPK30_SDS20%.

Figure 8 shows the dissolution profiles of CXB from SDs prepared with inulin, mannitol, and PVP K30. The PVP K30 based SD formulation was able to dissolve the highest amount of CXB and was therefore used for further optimization. It was visually observed that during dissolution PVP K30 formed a gel. Therefore, to promote disintegration, SDS was added to the formulation in different amounts. As can be seen in Figure 9, the addition of 10% and 20% of SDS resulted in an improved dissolution rate of CXB, indicated by the higher AUC. However, fast dissolution was followed by rapid precipitation. As the addition of HPMC 4000 cps was able to maintain the supersaturated state for the SEDDS formulation for a substantial period of time, this precipitation inhibitor was added to the formulation by co-freeze drying or by physical mixing with the freeze-dried powder. Unexpectedly, however, the presence of HPMC 4000 cps was unable to prevent precipitation (Figure 10). Also, HPMC 5 cps was unable to prevent precipitation of CXB. XRPD analysis showed that the formulations with PVPK30 and PVPK30_SDS20% were fully amorphous, see Figure 11. Modulated Differential Scanning Calorimetry experiments were also performed but it was impossible to confirm the amorphous state (data not shown). This was due to the fact that the melting temperature of CXB was higher than the onset glass transition temperature of PVPK30 (data not shown).

### 3.3. ColoPulse Coated Capsules

**Figure 12:**
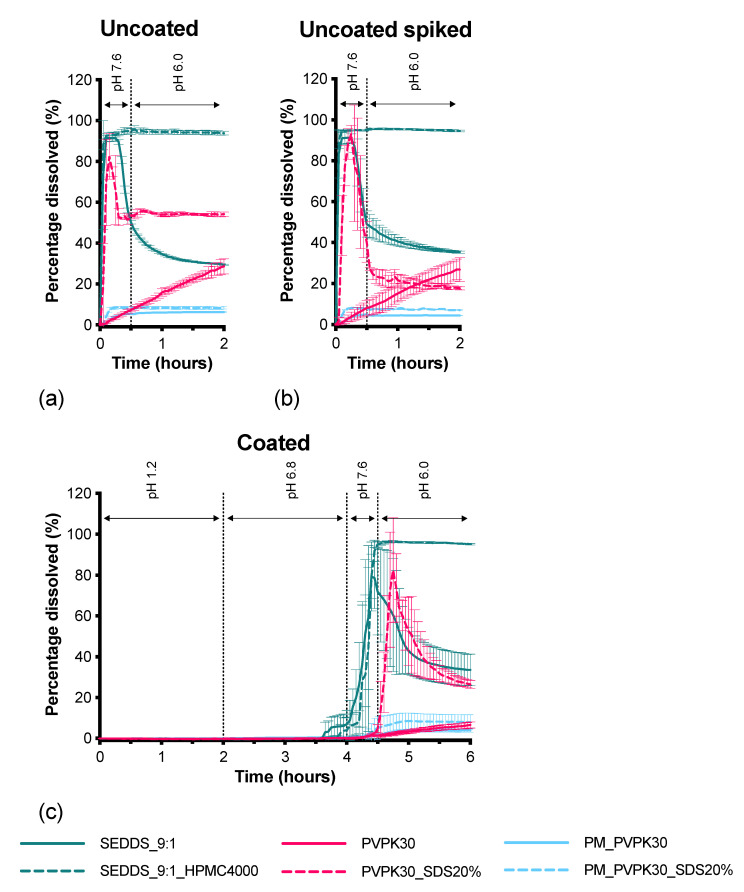
Influence of the ColoPulse coating on the release of CXB. (**a**) CXB release from uncoated capsules in GISS III-IV; (**b**) CXB release from uncoated capsules in GISS III-IV with ColoPulse suspension; (**c**) CXB release from coated capsules in GISS I-IV (mean ± SD, *n* = 3–4).

In Figure 12 the release profiles of the uncoated capsules in GISS phase III–IV (Figure 12a), the uncoated capsules in the presence of the ColoPulse coating in GISS phase III–IV (Figure 12b), and the ColoPulse coated capsules in GISS phase I–IV (Figure 12c) are given. These experiments were carried out to distinguish the influence of the components in the coating (comparison of Figure 12a,b) and the influence of the coating on the capsule (comparison of Figure 12a,c). The dissolution from the PVPK30_SDS20% formulation was negatively impacted by the presence of the ColoPulse components, both when present in the dissolution medium (Figure 12b) and when present in the form of a coating on the capsule (Figure 12c). In both cases, an ongoing decline in drug concentration was observed after the initial peak. Surprisingly, the PVPK30 formulation was not impacted by the presence of the ColoPulse components in the dissolution medium (Figure 12b). However, these dissolution profiles are far from ideal. Furthermore, when coated with the ColoPulse coating the release profile was considerably impacted and showed a similar release profile to the PM_PVPK30 formulation (Figure 12c).

The SEDDS_9:1 formulation was positively impacted by the presence of the ColoPulse coating in the dissolution medium (Figure 12b), although the effect was small. However, the coated capsules showed a slightly lower peak concentration (Figure 12c). The SEDDS_9:1_HPMC4000 formulation was not affected by the presence of the ColoPulse coating, neither when present in the dissolution medium (Figure 12b) nor when present on the capsule itself (Figure 12c). 

Overall, the SEDDS_9:1_HPMC4000 formulation was the only formulation that showed both the desired release profile for absorption in vivo, i.e., a high concentration for an extended period of time, and had a release profile that was not affected by the presence of a coating.

## 4. Discussion

Both the SEDDS and PVPK30 SD formulations could improve the dissolution behavior of CXB. The PM formulations on the other hand were unable to improve the dissolution of CXB, i.e., the maximum dissolved amount was within the range of the aqueous solubility of CXB at pH 7 (3–7 mg/mL) [5]. The optimized SEDDS formulation (SEDDS_9:1_HPMC4000) outperformed the SD formulations (PVPK30 and PVPK30_SDS20%). In addition, the ColoPulse coating did not affect the release profile of SEDDS_9:1_HPMC4000. Therefore, the ColoPulse coated SEDDS_9:1_HPMC4000 formulation could be beneficial for the treatment or prevention of colon cancer. 

To prevent precipitation of CXB different precipitation inhibitors were tested. PVPs of various molecular weights were unable to delay precipitation of CXB in the SEDDS_9:1 formulation (Figure 5), even though PVP K30 was found to be the best carrier for the SD formulation (Figure 8). Different HPMC grades on the other hand were able to delay precipitation of CXB in the SEDDS formulation (Figure 6). This polymer-induced delay in precipitation has previously been demonstrated in various SEDDS formulations [21]. For example, in an in vitro study the addition of HPMC to a SEDDS formulation could delay precipitation of paclitaxel [22]. In rats, this precipitation inhibition by HPMC lead to a 5-fold higher bioavailability. 

To prevent precipitation in the PVPK30 SD formulation it is important to know the physical state of CXB. XRPD analysis showed that the formulation was fully amorphous. This amorphous state could lead to a supersaturated state of CXB in the near vicinity of the dissolving capsule and could thus result in crystallization of CXB. Previously we have shown that incorporation of SDS in an SD by freeze-drying could prevent this crystallization [23]. Partially similar results were obtained in this study since incorporation of SDS resulted in a spring dissolution profile of CXB for the PVPK30_SDS20% formulation (Figure 9). However, the addition of SDS could not maintain the supersaturated state. The SDS concentration in the dissolution test was 0.15 mM which is less than the critical micelle concentration of SDS of 8 mM [24], thus no substantial effect on the saturation concentration of CXB was expected. In the SEDDS formulation, HPMC 4000 cps was found to be a good precipitation inhibitor. Unfortunately, co-freeze drying or physical mixing of HPMC 4000 cps into the PVPK30_SDS20% formulation did not prevent or delay precipitation (Figure 10). This discrepancy between the SEDDS and SD formulation is an indication that HPMC 4000 cps was incorporated into the emulsion droplets of the SEDDS formulation. If HPMC was present in the bulk of the dissolution medium during dissolution of the SEDDS, an influence on the dissolution profile of the SD formulation would be expected as well. Further, DLS analysis showed that the addition of HPMC altered the droplet size of the emulsion formed (Figure 7), indicating the accumulation of HPMC in these droplets, most likely at the oil-water interface. Similar results were found by Song et al. [7]. They showed that the addition of Soluplus to a SEDDS formulation of CXB had a stabilizing effect on the formed emulsion droplets. The Soluplus concentration in the formulation was found to be of importance since a too high amount could lead to steric hindrance or entanglement of the polymer leading to crystallization of the drug. Steric hindrance and entanglement of the polymers could also play a role when various types of HPMC are used (Figure 6). HPMCAS-LG has longer side chains than HPMC which could result in steric hindrance and prevent optimal precipitation inhibition [25]. HPMC 4000 cps with a medium molecular weight shows a better inhibitory effect on precipitation than HPMC 5 cps with a low molecular weight. According to Xu et al. this difference could be due to the increase in the number of available functional groups for interaction due to the higher molecular weight of HPMC 4000 cps [25]. However, viscosity could also have played a role. 

This study aimed to develop a formulation to deliver CXB to the colon for the local treatment or prevention of colon cancer. To obtain ileo-colonic delivery of CXB, the ColoPulse coating can be used. We found that the components in the ColoPulse coating (Figure 12b) and the application of the coating around the formulation (Figure 12c) may affect the dissolution behavior of the CXB formulations. The PVPK30_SDS20% formulation was negatively impacted by the presence of the coating, indicated by a larger CXB precipitation after the initial dissolution peak. This might be explained by the talc particles present in the dissolution medium which can act as nuclei, facilitating crystallization of CXB. Not only did the components in the ColoPulse coating have an impact on the release profile of CXB, but also on the physical inclusion of the formulation by the coating. The presence of the ColoPulse coating on the capsules had the greatest impact on the PVPK30 SD formulation. Its release profile resembled that of PM_PVPK30, indicating rapid crystallization of CXB. Even though SDS in the PVPK30_SDS20% formulation was unable to prevent crystallization, due to the presence of the nuclei in the coating, it was able to prevent the detrimental effect of inclusion. The inclusion affected the dissolution of the SEDDS_9:1 formulation as well, as indicated by a slightly lower peak concentration of CXB. In contrast, the dissolution behavior of the SEDDS_9:1_HPMC4000 formulation was unaffected. The results showed that the SEDDS formulations are best equipped to overcome the negative effect of the application of a coating, since the formulations can flow freely and quickly out of the capsules. Further nucleation crystals in the ColoPulse coating were unable to induce crystallization, probably due to the rapid formation of the emulsion.

In this study we were unable to develop an SD formulation that showed a similar dissolution behavior as the uncoated SEDDS_9:1_HPMC4000 formulation. An SD formulation with such dissolution behavior could have been less sensitive to the effects of a coating. Our results were obtained with only one drug and a limited number of formulations. Other drugs and formulations may behave differently. For instance, Sakai et al. showed that an HPMC coating improved the dissolution profile of an SD formulation of a poorly soluble drug (FK555) [26]. Another study showed that the in vitro release profile of a solid SMEDDS formulation of prednisolone was not impacted by a pH-dependent colon targeted film coating [27]. Overall, we hypothesize that in general, the liquid SEDDS formulations will outperform the SD formulations when a pH-dependent film coating is applied. Because coating systems generally do not disintegrate instantaneously, water can penetrate the systems before dispersion. In the case of the SEDDS formulations this will readily lead to the formation of an emulsion, which can freely flow out of the capsule. In the case of SD formulations, water penetration could lead to crystallization or crystal growth due to local supersaturation in the coated capsule. The addition of excipients such as SDS could prevent this crystallization. Furthermore, not only the components in the dissolution enabling systems should be selected with care. The components in the coating itself should also be taken into consideration since they can also affect the dissolution profile.

## 5. Conclusions

Overall, the CXB release profile of the SEDDS_9:1_HPMC4000 formulation was not negatively impacted by the physical obstruction and the components in the ColoPulse coating. However, the other formulations, SEDDS_9:1, PVPK30, and PVPK30_SDS20%, were negatively impacted by either the inclusion or the presence of nucleation crystals in the coating. This indicates that a SEDDS formulation that is not susceptible to crystallization works best in combination with the coating. Thus, a supersaturated SEDDS formulation with the ColoPulse coating could be beneficial for the treatment and prevention of colon cancer.

## Figures and Tables

**Table 1:** Specifications of the GISS, adapted with permission from [18], Eur. J. Pharm. Sci. 2007.

Phase	Segment Gastrointestinal Tract	pH	Volume (mL)	Time (h)
I	Stomach	1.20 ± 0.20	500	2.0
II	Jejunum	6.80 ± 0.20	629	2.0
III	Terminal ileum	7.63 ± 0.12	940	0.5
IV	Colon	6.00 ± 0.25	1000	1.5

**Table 2:** Composition of the SEDDS formulations with CXB.

Formulations	Composition				
	CXB (% *w*/*v*)	Precipitation Inhibitor (% *w*/*v*)	Tetraglycol (% *v*/*v*)	Cremophor RH40 (% *v*/*v*)	Capryol 90 (% *v*/*v*)
SEDDS_7:3_100	20		35	35	30
SEDDS_8:2_100	20		40	40	20
SEDDS_9:1_100	20		45	45	10
SEDDS_9:1	10		45	45	10
SEDDS_9:1_HPMC5	10	2	45	45	10
SEDDS_9:1_ HPMC4000	10	2	45	45	10
SEDDS_9:1_HPMCAS-LG	10	2	45	45	10
SEDDS_9:1_PVPK15	10	2	45	45	10
SEDDS_9:1_PVPK30	10	2	45	45	10
SEDDS_9:1_PVPK60	10	2	45	45	10
SEDDS_9:1_PVPK90	10	2	45	45	10

**Table 3:** Composition of the freeze-dried and physically mixed (PM) formulations with CXB.

Formulations	Composition (% *w*/*w*)							
	CXB	Inulin	Mannitol	PVP K30	Primojel^®^	SDS	HPMC 4000 cps	HPMC 5 cps
Inulin	22.5	73.5			4.0			
Mannitol	22.5		77.5					
PVPK30	22.5			73.5	4.0			
PVPK30_SDS10%	22.5			63.5	4.0	10		
PVPK30_SDS20%	22.5			53.5	4.0	20		
PVPK30_SDS20%_HPMC5PM	22.5			49	4.0	20		4.5 ^1^
PVPK30_SDS20%_HPMC5	22.5			53		20		4.5
PVPK30_SDS20%_HPMC4000PM	22.5			49	4.0	20	4.5 ^1^	
PVPK30_SDS20%_HPMC4000	22.5			53		20	4.5	
PM_PVPK30	22.5 ^2^			73.5 ^2^	4.0 ^2^			
PM_PVPK30_SDS20%	22.5 ^2^			53.5 ^2^	4.0 ^2^	20 ^2^		

^1^ Excipient was physically mixed with the SD instead of co-freeze dried. ^2^ CXB and excipients were physically mixed.

## Data Availability

Not applicable.
